# Effect of socioeconomic status on behavioral problems from preschool to early elementary school – A Japanese longitudinal study

**DOI:** 10.1371/journal.pone.0197961

**Published:** 2018-05-24

**Authors:** Rikuya Hosokawa, Toshiki Katsura

**Affiliations:** 1 School of Nursing, Nagoya City University, Nagoya, Japan; 2 Graduate School of Medicine, Kyoto University, Kyoto, Japan; TNO, NETHERLANDS

## Abstract

**Purpose:**

Social inequalities are widely accepted to have a deleterious effect on children’s mental health, and those with lower socioeconomic status generally experience more mental health issues. In this study, we examine the impact of socioeconomic situations of children’s families during their early childhood on the children’s social adaptation in Japanese elementary school.

**Methods:**

The current investigation consisted of two sets of data relating to two separate years (with a one-year interval). The participants included preschoolers aged five years at Time 1 (the first year) and first graders aged six years at Time 2 (the second year); 1,712 met the inclusion criteria for both years. Parents of the participants completed a self-reported questionnaire regarding their SES (i.e., family economy and mother’s education) and their children’s mental health. Mental health was assessed using the Child Behavior Checklist/4–18, Parent Report.

**Results:**

For each SES indicator, we found an inverse relationship across all the symptom dimensions. Specifically, bivariate analyses revealed that lower family income, maternal education level, and paternal education level predict all three domains of behavioral problems (i.e., internalized problems, externalized problems, and total behavioral problems). Further, multivariate analyses revealed that lower family income consistently predicts all domains of behavioral problems, lower maternal education level predicted externalized problems and total behavioral problems, and paternal education level did not predict any clinically significant behavioral problems.

**Conclusion:**

In this sample, we found that, for children, family income and parental education when entering preschool were significant predictors of mental health problems after elementary school enrollment; in particular, low income and low maternal educational achievement predicted a high probability of the development of a psychiatric disorder. A greater understanding of the mechanisms of these associations could contribute to improvements in interventions aimed at preventing child maladjustment.

## Introduction

It is widely accepted that social inequalities increase children’s risk of developing mental health problems [1–4]. In order to provide a better understanding of this, numerous studies on social inequalities and mental health have focused on low socioeconomic status (SES) as the main causal variable. SES is a concept that summarizes an individual’s social position in society. As previously demonstrated in a number of studies, socioeconomic position in terms of family economics and educational aspects as SES indicators has a strong influence on a child’s health and well-being [5–7]. Further, SES has been found to have a high likelihood of interacting with other factors, mediating or moderating their influence on children’s development. For example, lower SES can adversely influence children’s development in terms of behavioral and cognitive domains through several avenues, including lower levels of parental resources, social support, parental mental health, and parental functioning [8–10].

While studies on this topic have been quite common in certain geographic regions such as North America and several European countries, there have been limited attempts to examine the impact that indicators of families’ socioeconomic situations have on child development in Japan. The main reason indicators of families’ socioeconomic circumstances have rarely been studied in Japan is that information at the individual and household levels is relatively unavailable in the country. Consequently, most of the previous research on this topic has relied on retrospective reports concerning people’s childhood circumstances; however, such reports are regularly compromised by poor recall and measurement errors [11]. As socio-cultural backgrounds differ between countries, precisely clarifying the relationship between SES and childhood psychopathology in Japan could contribute to providing insight into the etiology of mental health problems and to improving interventions designed to reduce the burden of such mental health problems and prevent child maladjustment.

It is difficult to claim that Japan is a country that upholds social equality. Although Japan has no official poverty line, in practice, the relative poverty rate of the OECD index is frequently used to calculate the poverty rate. The threshold, calculated based on the OECD standard, which is half of the median income of the total population, was defined as approximately 1.2 million JPY [12]. Japan’s relative poverty rates are higher than the OECD average, and are steadily increasing [13]; in particular, poverty among young adults and families with children has shown a marked increase [14–16]. Notably, Japan is in the top third of OECD countries in terms of levels of average family income, but some children in the country do not enjoy the benefits of this position; specifically, child poverty in Japan, at approximately 14%, is higher than the OECD average of roughly 12%. In addition, the relative poverty rate among single-parent households in Japan is over 50%, which is the highest of all OECD nations. Concurrently, concerns in regard to the potential transmission of poverty from parents to children have become heightened in Japan, mainly fueled by the country’s widening income inequality and increasing poverty risks [17]. From a global perspective, absolute poverty is decreasing, but in the developed world, divides between the poor and the wealthy are widening [14–16]; thus, relative child poverty is becoming a central issue that must be addressed not only in Japan but also in many developed countries.

Although absolute levels of affluence are critical variables for predicting child development, to better predict various social and behavioral health outcomes, an increasing emphasis is being placed on relative levels of affluence [18]. Several studies have suggested that relative deprivation, which is defined as individual SES relative to that of one’s peers in society, might have a closer relationship with child behavioral symptoms than absolute difference in income has with such symptoms [19,20]. In countries with high relative social inequality, such as Japan, people with lower SES may have lower levels of happiness across many dimensions than people with higher SES. Further, feelings of relative deprivation may contribute to worsening the conditions of people with lower SES, because they can be motivated to spend lavishly on goods and services that signify higher status in society. Such stressful experiences may outweigh the few existing parental resources, resulting in maladaptive coping mechanisms that foster conflict among family members and disrupted child-rearing skills, consequently predicting mental health problems in children. Thus, relative inequality, which is measured by analyzing the resources needed to maintain a particular social and economic lifestyle, is an important but often-neglected policy target. Therefore, in order to understand the mechanism of SES and child development, it is important to clarify the relationship between SES and children’s development in Japan.

On the other hand, many studies have measured SES by combining education status and other SES variables to create simple composite scores [21]. However, while several of these studies have found that lower parental education is related to lower levels of developmental outcomes in children, including psychological well-being and emotional and cognitive development [22–25], there is limited evidence that parental education, as an SES indicator, independently predicts child developmental outcomes. Japan is a country with high educational standards: access to education is high across all levels, enrollment in pre-primary education is high, and first-time entry and graduation rates at the tertiary level are also high [26,27]. Further, roughly half of the working-age population in Japan is tertiary-educated, which is much higher than the OECD average of approximately 35%. In addition, based on current patterns of graduation, over 70% of young people in Japan are expected to graduate from tertiary education during their lifetimes, and this is also higher than the OECD average of approximately 50%. On the other hand, regarding the relationship between education and employment, approximately 80% of tertiary-educated adults in Japan are employed, while less than 75% of adults with lower levels of education have jobs. Concurrently, the unemployment rate among tertiary-educated adults decreased between 2000 and 2012 from 3.5% to 3.2%, while that of adults with upper secondary or post-secondary non-tertiary education increased from 4.7% to 5.1% during the same period. This shift means that the gap between adults with high and low levels of education has widened over the past 12 years. As shown by these statistics, the social inequalities resulting from parental educational achievement might negatively influence parents’ mental health and result in poor child developmental outcomes.

The effects of socioeconomic circumstances are likely to vary depending on the phase of life in which they are experienced. In particular, prior studies investigating this topic have suggested that such effects are most pronounced during preschool years. The reason for this may be that poverty interferes with the processes that lead to school readiness. Moreover, childhood SES is not the only aspect that has an impact on developmental outcomes and mental health in childhood, as hardships during childhood are also likely to be critical for explaining differences in developmental outcomes and mental health later in life. For instance, exposure to poverty during childhood appears to have a greater detrimental impact on childhood social and cognitive ability than experiencing poverty later in life [28–31]. In addition, childhood SES has been found to be a relevant predictor of adult mental health status [32,33]. Furthermore, cumulative adversities experienced in childhood have also been found to be associated with psychological distress in adulthood [34–36]; in other words, persistent economic deprivation appears to be more detrimental than transient poverty. Mental health problems experienced during childhood are known to be associated with both current and later impairment, and to result in maladjustment [37,38]. This realization has led to the development of numerous early childhood intervention programs, such as “Head Start,” targeting low-SES children, and this has been proven to be effective for promoting children’s development [39–41]. Considering the above findings cumulatively, it seems that adversity in childhood becomes embodied at an early age, and the full impact of this then manifests itself later in adult life. Therefore, experiencing adversity and social inequalities early in life is likely to be more detrimental than experiencing them at any later point. Consequently, it is important to verify that this is the case: social inequalities in early childhood have a severe impact on development later in life.

In the current research, using a longitudinal design, we examined the impact SES in early childhood has on social adaptation in the first grade of elementary school in Japan. To achieve this, we included three different indicators of SES (i.e., family economy, paternal education level, and maternal education level). In addition, we included an assessment of mental health, including behavioral problems, in regard to the child’s everyday functioning. Consequently, our investigation was successful, and we determined that SES, particularly lower family income and lower maternal education, is a predictor of future behavioral problems. We anticipate that the results of this research will help inform policies and plans that support the development of children in countries with high relative poverty rates.

## Methods

### Participants

The current investigation consists of two sets of data relating to two different years (with a one-year interval between them) and is part of a longitudinal study examining the influence of family factors on children’s social developmental outcomes. At Time 1 (T1), which was conducted in 2014, we obtained a sample of preschool children, all of whom were five years old, from those enrolled in 52 kindergartens and 78 nursery schools in Nagoya City, which is a major urban area in Japan. To recruit these participants, self-reported questionnaires were distributed to all parents of targeted children (*n* = 5,024), and the parents were asked to complete the questionnaires (number of responses, *n* = 3,314). Then, at Time 2 (T2), which was conducted in 2015, the same children were recruited, now six years old and in the first grade. In other words, a similar questionnaire was provided to parents one year (12 months) after T1. Once again, the parents completed the questionnaires (number of responses, *n* = 1,787). The retention rate from T1 to T2 was 53.9%, meaning attrition from T1 to T2 was 46.1%, with 44.7% not responding at T2 and 1.4% (*n* = 46) having relocated. Comparing the non-returning participants with the returning participants on demographic features, regarding household income, 16.2% of the non-returning participants’ household income per year was below Ұ 2,999,999, while this was true of only 10.0% of returning participants. The household income of the non-returning participants was significantly lower than that of the returning participants, as measured by a chi-square test. In addition, regarding education level, 5.8% of the non-returning participants’ maternal educational background was compulsory education, while this was true of only 2.4% of returning participants. Furthermore, 7.6% of the non-returning participants’ paternal educational background was compulsory education, while this was true of only 4.7% of returning participants; both maternal and paternal education levels of non-returning participants were significantly lower than those of the returning participants, as measured by a chi-square test. Thus, the non-returning participants tended to have relatively lower SES (i.e., family income and parental education level) than did returning participants, meaning that there was a lower response rate for individuals with low SES compared to high SES. To accurately clarify the associations between SES and child developmental outcomes, we then chose to exclude children with developmental problems. Consequently, of the 1,787 children for whom data was received at both T1 and T2, 1,712 (95.8%) met the inclusion criteria.

### Ethics statement

Researchers obtained written, informed consent from all participants. For the children, written, informed consent was obtained from parents on their behalf. Ethical approval for this study was obtained from Kyoto University’s Ethics Committee in Kyoto, Japan (E2322).

### Measurements

#### Outcome variable: Child behavioral problems

Behavioral problems were assessed using the Child Behavior Checklist/4–18 (CBCL), Parent Report, which targets children aged 4 to 18 years. [42]. For this study, the Japanese Edition of the CBCL was used, which consists of 113 items [43]. Each item was rated using a three-point scale ranging from 0 (“not true”) to 2 (“very true” or “often true”). Ratings for subsets of items were then summed to provide scores for the eight syndrome scales (i.e., withdrawn, somatic complaint, anxious/depressed, social problems, thought problems, attention problems, delinquent behavior, and aggressive behavior). Then, the scores of the syndrome scales were combined to determine scores for the higher-order domains of internalized, externalized, and total problems. Internalized factors include items relating to withdrawal, somatic complaints, and anxiety/depression, while externalized factors include items assessing delinquency and aggression. Next, the T-scores of the CBCL’s internalized, externalized, and total problem scores were calculated using standardized distribution among Japanese children, and T-scores greater than or equal to 63 in the internalized, externalized, and total problem scales were defined as indicating clinically significant “abnormal” symptoms [43]. The scale has been well standardized, and various studies have found it to have good reliability and validity [44,45]. In the current study, Cronbach’s α coefficient was found to be adequate, with the value for the externalized behaviors being .88, for internalized behaviors being .87, and for total behavior problems being .89.

#### Explanatory variable: Socioeconomic status

In the next stage, SES was defined using information concerning family income and parental education. First, parents reported their annual equalized household income in Japanese Yen (JPY). Then, using this data, we created four categories of income: <3,000,000 JPY (approximately 30,000 USD), 3–4,000,000 JPY (approximately 30,000–40,000 USD), 5–6,000,000 JPY (approximately 50,000–60,000 USD), and ≥7,000,000 JPY (approximately 70,000 USD). Further, both parents of each child were also asked to report their education in years, as well as the highest educational level completed, using the following choices: elementary school (six years), junior high school (nine years), vocational or general upper secondary school (12 years), less than four years at college/university (13–15 years), four years at college/university (16 years), and graduate school (>16 years). It should be noted here that the Japanese education system comprises elementary school (six years), junior high school (three years), and high school (three years), and education is compulsory until the end of junior high school (nine years). Based on this data, we created four categories indicating education level: compulsory education (nine years), upper secondary school (12 years), up to four years of college/university (13–15 years), and over four years of college/university (>15 years).

#### Covariates: Demographic information

Parents also provided background demographic information for their children, from which covariates were sourced. This information included sex, family composition (nuclear or expanded family), family status (two parents or single parent), number of siblings (one or more siblings or no siblings), and preschool institution attended (kindergarten or nursery school).

#### Data analyses

Poisson regression analyses were conducted to assess the association between SES and diagnostic probability scores; this was due to the high prevalence of the outcomes (i.e., the CBCL clinically significant behavioral problems) [46–48]. The dichotomized probability score for predicted behavioral problems was then entered as the dependent variable (non-abnormal = 0 and abnormal = 1), and family income and paternal and maternal education levels were entered as categorical predictors. Specifically, the associations between the children’s characteristics and SES at preschool and between SES and behavioral problems in the first grade were analyzed using a bivariate Poisson regression model; then, multivariate Poisson regression using significant variables identified from the bivariate regression was used to examine the independent associations between SES and behavioral problems. In the multivariate model, as the demographic factors were not significantly associated with behavioral problems in the bivariate analyses, we did not include the demographic factors in the multivariate analyses. On the other hand, as all socioeconomic status factors were significantly associated with behavioral problems in the bivariate analyses, we included all socioeconomic status factors in the analyses. More specifically, although not all indicators were significantly related to behavioral problems in the bivariate analyses, all indicators of annual household income, maternal education level, and paternal education level were covaried in the multivariate analyses.

In addition, to investigate how variables related to each other, we conducted path analyses; the path analyses were conducted to estimate direct and indirect paths between family income, maternal and paternal education levels, and behavioral problems (*see S2 Fig, S3 Fig, and S4 Fig*). The hypothesized model is presented in S1 Fig; in the model, maternal and paternal education levels were specified as predictors of family income and child behavioral problems. Prior to conducting the path analyses, correlational analyses were performed to measure associations between demographic variables and outcome variables; variables significantly correlated with total behavioral problems were entered into the predictive model as control variables (*see S1 Table*).

All statistical analyses were conducted using SPSS Statistics 23.0 and AMOS version 23.0.

## Results

### Study population

Table 1 shows the demographic characteristics of children, familial SES at T1, and the prevalence of clinically significant behavioral problems, which was determined using the CBCL, at T2. At T1, the children’s mean age was 6.09 years (SD = 0.30), and the sex distribution was almost equal. Additionally, 86.4% lived in a nuclear family, 93.7% lived with both parents, 83.2% had one or more siblings, and the distribution of preschool institution attended (kindergarten or nursery school) was almost equal. Additionally, the mean ages of the mothers and fathers were 37.28 (SD = 4.62) and 39.29 (SD = 5.45) years, respectively. In the Japanese population, approximately 80% lived in a nuclear family, approximately 95% lived with both parents, and approximately 80% had one or more siblings [12]. Therefore, participants in the current study were roughly similar to the demographics of the Japanese population. Regarding SES, the median household income was between 5,000,000 and 5,999,999 JPY per year, and the ratio of families with an annual income of less than 3,000,000 JPY was 10.0%. Next, in relation to parents’ education status, the most common level of educational achievement for mothers was less than four years at college/university (40.7%), while the most common paternal educational achievement was having attended over four years of college/university (53.3%: undergraduate degree (four years); 45.9%, graduate degree (>four years); 7.4%). Meanwhile, the percentage of compulsory education (nine years) was 2.4% for mothers, while for fathers it was 4.7%; on the other hand, the proportion of having attended over four years at college/university for mothers was 32.1%, while for fathers it was 53.3%. In terms of Japanese SES, among a similar generation, the median household income was approximately 4.5 million JPY [12]. The proportion of having attended over four years of college/university for women was approximately 20% and for men was approximately 35% [49]. Therefore, SES of participants in the current study was relatively higher compared with that typical of the Japanese. Finally, regarding the CBCL results for clinically significant behavioral problems, the clinical cut-off was exceeded for internalized, externalized, and total behavior problems in 17.8%, 17.6%, and 18.8% of participants, respectively.

**Table 1:** Demographic characteristics and socioeconomic status of subjects at preschool, as well as CBCL behavioral problems in first grade (N = 1,712).

			*n*	*%*
Demographics	Child sex			
		Female	825	48.2
		Male	887	51.8
	Family composition			
		Nuclear family	1480	86.4
		Expanded family	232	13.6
	Family status			
		Two parents	1604	93.7
		One parent	108	6.3
	Number of siblings			
		One or more siblings	1425	83.2
		No siblings	287	16.8
	Preschool institution attended			
		Kindergarten	808	47.2
		Nursery school	904	52.8
Socioeconomic status	Annual household income (in million JPY)			
		≥ 7	547	32.0
		5–6	515	30.1
		3–4	434	25.4
		< 3	172	10.0
		No response	44	2.6
	Maternal education level			
		Over four years at college/university (≥ 16 years)	549	32.1
		Less than four years at college/university (13–15 years)	696	40.7
		Upper secondary school (12 years)	409	23.9
		Compulsory education (nine years)	41	2.4
		No response	17	1.0
	Paternal education level			
		Over four years at college/university (≥ 16 years)	913	53.3
		Less than four years at college/university (13–15 years)	243	14.2
		Upper secondary school (12 years)	398	23.2
		Compulsory education (nine years)	81	4.7
		No response	77	4.5
CBCL clinically significant behavioral problems				
	Internalized behavioral problems		304	17.8
	Externalized behavioral problems		301	17.6
	Total behavioral problems		322	18.8

CBCL: Child Behavior Checklist.

### Internalized behavioral problems

Associations of demographic characteristics and SES with clinically significant internalized behavioral problems are shown in Table 2. In our bivariate model, when children from families with an annual household income over 7,000,000 JPY were used as the reference category, we found that annual household income showed a significant association with clinically significant internalized behavioral problems (3–4,000,000: rate ratio (RR): 1.83, 95% confidence interval (CI): 1.35–2.50; less than 3,000,000: RR: 2.06, 95% CI: 1.41–3.01), and this association remained significant in the multivariate model, which was mutually adjusted. (In the multivariate model, as the demographic factors were not significantly associated with internalized behavioral problems in the bivariate analyses, we did not include demographic factors in the analyses. On the other hand, as all socioeconomic status factors were significantly associated with internalized behavioral problems in the bivariate analyses, we included all socioeconomic status factors in the multivariate analyses. More specifically, although not all indicators were significantly related to internalized behavioral problems in the bivariate analyses, all indicators of annual household income, maternal education level, and paternal education level were covaried in the multivariate analyses.) In other words, the rate ratio suggests that children from families with an annual household income of 3 to 4,000,000 JPY and less than 3,000,000 JPY were 1.66 times (95% CI: 1.20–2.30) and 1.91 times (95% CI: 1.24–2.94) more likely to show clinically significant internalized problems, respectively, independent of other SES indicators, compared to children from families with an annual household income over 7,000,000 JPY.

**Table 2:** Bivariate and multivariate analysis predicting CBCL clinically significant internalized behavioral problems (N = 1,712).

			CBCL clinically significant internalized behavioral problems							
			Abnormality rate		Bivariate model			Multivariate model		
			*n*	*%*	*RR*	*95% CI*	*p*	*RR*	*95% CI*	*p*
Demographics	Child sex									
		Female	145	17.6	Ref					
		Male	159	17.9	1.02	(0.81–1.28)	.864			
	Family composition									
		Nuclear family	259	17.5	Ref					
		Expanded family	45	19.4	1.11	(0.81–1.52)	.524			
	Family status									
		Two parents	282	17.6	Ref					
		One parent	22	20.4	1.16	(0.75–1.79)	.506			
	Number of siblings									
		One or more siblings	252	17.7	Ref					
		No siblings	52	18.1	1.03	(0.78–1.36)	.835			
	Preschool institution attended									
		Kindergarten	144	17.8	Ref					
		Nursery school	160	17.7	0.99	(0.79–1.24)	.952			
Socioeconomic status	Annual household income (in million JPY)									
		≥ 7	68	12.4	Ref			Ref		
		5–6	86	16.7	1.34	(0.98–1.85)	.069	1.28	(0.93–1.77)	.129
		3–4	99	22.8	1.83	(1.35–2.50)	.000	1.66	(1.20–2.30)	.002
		< 3	44	25.6	2.06	(1.41–3.01)	.000	1.91	(1.24–2.94)	.002
		No response	7	15.9	1.28	(0.59–2.79)	.534	1.22	(0.56–2.70)	.616
	Maternal education level									
		Over four years at college/university (≥ 16 years)	74	13.5	Ref			Ref		
		Less than four years at college/university (13–15 years)	121	17.4	1.29	(0.97–1.72)	.085	1.18	(0.87–1.59)	.291
		Upper secondary school (12 years)	94	23.0	1.71	(1.26–2.31)	.001	1.37	(0.97–1.94)	.077
		Compulsory education (nine years)	12	29.3	2.17	(1.18–4.00)	.013	1.61	(0.81–3.32)	.176
		No response	3	17.6	1.31	(0.41–4.15)	.647	1.36	(0.40–4.59)	.621
	Paternal education level									
		Over four years at college/university (≥ 16 years)	142	15.6	Ref			Ref		
		Less than four years at college/university (13–15 years)	45	18.5	1.19	(0.85–1.66)	.308	1.00	(0.65–1.33)	.697
		Upper secondary school (12 years)	81	20.4	1.31	(1.00–1.72)	.053	1.04	(0.77–1.39)	.818
		Compulsory education (nine years)	22	27.2	1.75	(1.11–2.74)	.015	1.21	(0.74–1.97)	.456
		No response	14	18.2	1.17	(0.68–2.02)	.577	0.73	(0.39–1.38)	.334

CBCL: Child Behavior Checklist.

When children of parents with over four years at college/university (≥ 16 years) were used as the reference category, although we found in the bivariate model that maternal education level showed a significant association with clinically significant internalized behavioral problems (upper secondary school (12 years): RR: 1.71, 95% CI: 1.26–2.31; compulsory education (nine years): RR: 2.17, 95% CI: 1.18–4.00), no significant association was found in the multivariate model. Similarly, although we found that paternal education level showed a significant association with clinically significant internalized behavioral problems in the bivariate model (compulsory education (nine years): RR: 1.75, 95% CI: 1.11–2.74), no significant association was found in the multivariate model.

In addition, to estimate how these variables related to each other, we conducted path analyses to estimate the direct and indirect paths between family income, maternal and paternal education levels, and internalized behavioral problems (*see S2 Fig*). The result of the analyses showed that maternal and paternal education levels were indirectly related to internalized behavioral problems through family income; on the other hand, maternal and paternal education levels were not directly related to internalized behavioral problems.

### Externalized behavioral problems

The associations of demographics and SES with clinically significant externalized behavior problems are shown in Table 3. In our bivariate model, when children from families with an annual household income over 7,000,000 JPY were used as the reference category, we found that annual household income showed a significant association with clinically significant externalized behavioral problems (3–4,000,000: RR: 1.39, 95% CI: 1.02–1.91; less than 3,000,000: RR: 1.74, 95% CI: 1.19–2.54), and this association remained significant in the multivariate model. (In the multivariate model, as the demographic factors were not significantly associated with externalized behavioral problems in the bivariate analyses, we did not include demographic factors in the analyses. On the other hand, as all socioeconomic status factors were significantly associated with externalized behavioral problems in the bivariate analyses, we included all socioeconomic status factors in the multivariate analyses; more specifically, although not all indicators were significantly related to externalized behavioral problems in the bivariate analyses, all indicators of annual household income, maternal education level, and paternal education level were covaried in the multivariate analyses.) That is, the rate ratio suggests that children from families with an annual household income of less than 3,000,000 JPY were 1.54 times (95% CI: 1.02–2.42) more likely to show clinically significant externalized problems, independent of other SES indicators, compared to children from families with an annual household income of over 7,000,000 JPY.

**Table 3:** Bivariate and multivariate analysis predicting CBCL clinically significant externalized behavioral problems (N = 1,712).

			CBCL clinically significant externalized behavioral problems							
			Abnormality rate		Bivariate model			Multivariate model		
			*n*	*%*	*RR*	*95% CI*	*p*	*RR*	*95% CI*	*p*
Demographics	Child sex									
		Female	140	17.0	Ref					
		Male	161	18.2	1.07	(0.85–1.34)	.560			
	Family composition									
		Nuclear family	259	17.5	Ref					
		Expanded family	42	18.1	1.03	(0.75–1.43)	.839			
	Family status									
		Two parents	277	17.3	Ref					
		One parent	24	22.2	1.29	(0.85–1.95)	.236			
	Number of siblings									
		One or more siblings	245	17.2	Ref					
		No siblings	56	19.5	1.14	(0.85–1.52)	.393			
	Preschool institution attended									
		Kindergarten	134	16.6	Ref					
		Nursery school	167	18.5	1.11	(0.89–1.40)	.352			
Socioeconomic status	Annual household income (in million JPY)									
		≥ 7	75	13.7	Ref			Ref		
		5–6	93	18.1	1.32	(0.97–1.79)	.076	1.24	(0.91–1.69)	.165
		3–4	83	19.1	1.39	(1.02–1.91)	.037	1.28	(0.92–1.79)	.145
		< 3	41	23.8	1.74	(1.19–2.54)	.004	1.54	(1.02–2.42)	.044
		No response	9	20.5	1.49	(0.75–2.98)	.257	1.35	(0.66–2.74)	.412
	Maternal education level									
		Over four years at college/university (≥ 16 years)	80	14.6	Ref			Ref		
		Less than four years at college/university (13–15 years)	119	17.1	1.17	(0.88–1.56)	.269	1.09	(0.81–1.47)	.554
		Upper secondary school (12 years)	83	20.3	1.39	(1.02–1.89)	.035	1.22	(0.87–1.72)	.249
		Compulsory education (nine years)	16	39.0	2.68	(1.57–4.58)	.000	2.08	(1.14–3.79)	.016
		No response	3	17.6	1.21	(0.38–3.84)	.745	0.98	(0.29–3.37)	.974
	Paternal education level									
		Over four years at college/university (≥ 16 years)	141	15.4	Ref			Ref		
		Less than four years at college/university (13–15 years)	48	19.8	1.28	(0.92–1.77)	.141	1.11	(0.79–1.58)	.527
		Upper secondary school (12 years)	73	18.3	1.19	(0.90–1.58)	.233	1.04	(0.76–1.41)	.817
		Compulsory education (nine years)	21	25.9	1.68	(1.06–2.66)	.027	1.25	(0.75–2.07)	.396
		No response	18	23.4	1.51	(0.93–2.47)	.098	1.18	(0.65–2.12)	.593

CBCL: Child Behavior Checklist.

Similarly, in our bivariate model, when children from parents with over four years at college/university (≥ 16 years) were used as the reference category, we found that maternal education level showed a significant association with clinically significant externalized behavioral problems (upper secondary school (12 years): RR: 1.39, 95% CI: 1.02–1.89; compulsory education (nine years): RR: 2.68, 95% CI: 1.57–4.58), and this association remained significant in the multivariate model. In other words, the rate ratio suggests that children from families in which the maternal education level is compulsory education (nine years) were 2.08 times (95% CI: 1.14–3.79) more likely to show clinically significant externalized problems, independent of other SES indicators, compared to children from families with a maternal education level of over four years at college/university (≥ 16 years). On the other hand, although we found in the bivariate model that paternal education level shows a significant association with clinically significant externalized behavioral problems (compulsory education (nine years): RR: 1.68, 95% CI: 1.06–2.66), no significant association was found in the multivariate model.

In addition, to investigate how these variables related to each other, we performed path analyses, which were conducted to estimate direct and indirect paths between family incomes, maternal and paternal education levels, and externalized behavioral problems (*see S3 Fig*). The result of the analyses showed that paternal and maternal education level indirectly related to externalized behavioral problems through family income; at the same time, maternal education level directly related to externalized behavioral problems, which was mutually adjusted.

### Total behavioral problems

Finally, associations of demographics and SES with clinically significant total behavioral problems are shown in Table 4. In our bivariate model, when children from families with an annual household income over 7,000,000 JPY were used as the reference category, we found that annual household income showed a significant association with clinically significant total behavioral problems (5–6,000,000: RR: 1.47, 95% CI: 1.08–1.99; 3–4,000,000: RR: 1.78, 95% CI: 1.31–2.41; less than 3,000,000: RR: 2.11, 95% CI: 1.46–3.04), and this association remained significant in the multivariate model. (In the multivariate model, as the demographic factors were not significantly associated with total behavioral problems in the bivariate analyses, we did not include demographic factors in the analyses. On the other hand, as all socioeconomic status factors were significantly associated with total behavioral problems in the bivariate analyses, we included all socioeconomic status factors in the multivariate analyses; more specifically, although not all indicators were significantly related to total behavioral problems in the bivariate analyses, all indicators of annual household income, maternal education level, and paternal education level were covaried in the multivariate analyses.) That is, the rate ratio suggested that children from families with an annual household income of 3–4,000,000 and less than 3,000,000 JPY are 1.55 times (95% CI: 1.12–2.13) and 1.72 times (95% CI: 1.13–2.63) more likely, respectively, to show clinically significant total behavioral problems, independent of other SES indicators, than children from families with an annual household income of over 7,000,000 JPY.

**Table 4:** Bivariate and multivariate analysis predicting CBCL clinically significant total behavioral problems (N = 1,712).

			CBCL clinically significant total behavioral problems							
			Abnormality rate		Bivariate model			Multivariate model		
			*n*	*%*	*RR*	*95% CI*	*p*	*RR*	*95% CI*	*p*
Demographics	Child sex									
		Female	148	17.9	Ref					
		Male	174	19.6	1.09	(0.88–1.36)	.424			
	Family composition									
		Nuclear family	275	18.6	Ref					
		Expanded family	47	20.3	1.09	(0.80–1.49)	.584			
	Family status									
		Two parents	297	18.5	Ref					
		One parent	25	23.1	1.25	(0.83–1.88)	.284			
	Number of siblings									
		One or more siblings	266	18.7	Ref					
		No sibling	56	19.5	1.04	(0.79–1.35)	.788			
	Preschool institution attended									
		Kindergarten	146	18.1	Ref					
		Nursery school	176	19.5	1.08	(0.87–1.34)	.505			
Socioeconomic status	Annual household income (in million JPY)									
		≥ 7	71	13.0	Ref			Ref		
		5–6	98	19.0	1.47	(1.08–1.99)	.014	1.36	(1.00–1.86)	.050
		3–4	100	23.0	1.78	(1.31–2.41)	.000	1.55	(1.12–2.13)	.008
		< 3	47	27.3	2.11	(1.46–3.04)	.000	1.72	(1.13–2.63)	.012
		No response	6	13.6	1.05	(0.46–2.42)	.908	0.95	(0.41–2.22)	.912
	Maternal education level									
		Over four years at college/university (≥ 16 years)	76	13.8	Ref			Ref		
		Less than four years at college/university (13–15 years)	131	18.8	1.36	(1.02–1.80)	.033	1.22	(0.91–1.64)	.182
		Upper secondary school (12 years)	94	23.0	1.66	(1.23–2.25)	.001	1.35	(0.97–1.89)	.076
		Compulsory education (nine years)	18	43.9	3.17	(1.90–5.30)	.000	2.28	(1.28–4.04)	.005
		No response	3	17.6	1.28	(0.40–4.04)	.680	1.21	(0.36–4.07)	.757
	Paternal education level									
		Over four years at college/university (≥ 16 years)	143	15.7	Ref			Ref		
		Less than four years at college/university (13–15 years)	51	21.0	1.34	(0.97–1.82)	.073	1.07	(0.77–1.50)	.677
		Upper secondary school (12 years)	87	21.9	1.40	(1.07–1.84)	.014	1.12	(0.84–1.50)	.440
		Compulsory education (nine years)	24	29.6	1.89	(1.23–2.91)	.004	1.26	(0.78–2.03)	.341
		No response	17	22.1	1.41	(0.85–2.33)	.181	0.96	(0.53–1.73)	.895

CBCL: Child Behavior Checklist.

Similarly, in our bivariate model, when children of parents with over four years at college/university (≥ 16 years) were used as the reference category, we found that maternal education level showed a significant association with clinically significant externalized behavioral problems (less than four years at college/university (13–15 years): 1.36, 95% CI: 1.02–1.89; upper secondary school (12 years): RR: 1.66, 95% CI: 1.23–2.25; compulsory education (nine years): RR: 3.17, 95% CI: 1.90–5.30), and this remained significant in the multivariate model; that is, the rate ratio suggests that children from families with a maternal education level of compulsory education (nine years) were 2.14 times (95% CI: 1.16–3.97) more likely to show clinically significant externalized problems, independent of other SES indicators, compared to children from families with a maternal education level of over four years at college/university (≥ 16 years). On the other hand, although we found in the bivariate model that paternal education level showed a significant association with clinically significant externalized behavioral problems (upper secondary school (12 years): RR: 1.40, 95% CI: 1.07–1.84; compulsory education (nine years): RR; 1.89, 95% CI: 1.23–2.91), no such significance was observed in the multivariate model.

In addition, to investigate how these variables related to each other, we conducted path analyses to estimate the direct and indirect paths between family income, maternal and paternal education levels, and total behavioral problems (*see S4 Fig*). The results of the analyses showed that paternal and maternal education level was indirectly related to total behavioral problems through family income; at the same time, maternal education level was directly related to total behavioral problems, which was mutually adjusted.

## Discussion

In the present study, we found that lower SES at the preschool level negatively influenced behavioral problems upon entering school to a clinically significant degree, suggesting that children from families with lower SES have a higher risk of developing behavioral problems. Specifically, regarding family income, bivariate analyses revealed that lower family income significantly influenced all domains of clinical behavioral problems (i.e., internalized problems, externalized problems, and total behavioral problems). Furthermore, multivariate analyses also revealed that lower family income had a clinically significant influence within all of these domains of behavioral problems.

Similarly, regarding education level, bivariate analyses revealed that lower maternal and paternal education level significantly influence all domains of clinical behavioral problems (i.e., internalized problems, externalized problems, and total behavioral problems). Further multivariate analyses revealed that lower maternal education level negatively influenced externalized problems and total behavioral problems to a clinically significant degree while multivariate analyses revealed that paternal education level does not influence any behavioral problems to a clinically significant degree. These results are consistent with previous findings that lower familial socioeconomic circumstances during childhood influences the development of mental health problems later in life [1–4].

There are several potential mechanisms through which SES might influence the occurrence of behavioral problems. For example, many studies have suggested that social inequalities influence child development through not only the direct material path (which concerns an unequal distribution of material resources that can be used to support healthy child development [35,50]), which reflects material deprivation, but also through the indirect psychosocial path influenced by relative socioeconomic position [18,51,52]. That is, lower SES is likely to negatively affect developmental outcomes in children, as parents in such circumstances are unable to provide material resources necessary for healthy child development. On the other hand, lower SES is also likely to negatively affect developmental outcomes in children because these parents experience psychological distress; lower SES is associated with poor parental mental health, which in turn negatively influences parental functioning and parent–child interactions, predicting mental health problems in children [53].

In the current study, regarding family income, in the multivariate model, 3–4 million JPY was significantly related to internalized behavioral problems and total behavioral problems; in addition, <3 million JPY was significantly related to all of the domains of behavioral problems (i.e., internalized problems, externalized problems, and total behavioral problems). As mentioned earlier, in terms of Japanese SES, among similar generation families, the median household income was approximately 4.5 million JPY [12]. Children in families earning an income of <4 million JPY might be more strongly affected by relative poverty, than children of family’s earning more than the median household income.

Regarding parental educational level, in the multivariate model, maternal education level of compulsory education was significantly related to externalized problems and total behavioral problems. In Japan, the upper secondary school admission rate exceeds more than 95% [49]. Children of mothers completing only compulsory education (i.e., not completing more than upper secondary school) might be more strongly affected by educational inequality, than children of mothers completing more than upper secondary school. Therefore, social inequalities might influence child behavioral problems through not only the direct path but also through the indirect path influenced by relative socioeconomic position.

When considering the impact of family income on child outcomes, many parents in stressful economic situations are unable to provide the tangible or intangible resources necessary to support the successful development of their children [54,55]. One of the most critical pathways in this regard is the quality of the home environment. Lower SES is likely to create a family environment that is unsuitable for children’s healthy development, consequently affecting children’s developmental outcomes. Several studies have suggested that differences in the qualities of the home environments of higher- and lower-income children account for a substantial portion of the effect family income-to-needs has on the development of children [35,56,57].

Another important viewpoint is the investment perspective. Family income dictates the amount of time and money that families invest in materials and experiences that foster children’s development [50]. Families that are more financially secure are better able to invest time enriching their children, and they have more money to spend on helping their children experience greater opportunities to stimulate learning, on opportunities to encourage talent, and on improving the physical condition of their homes [58]. In other words, higher levels of income are likely to be associated with greater access to better home environments, such as reading materials and toys, cultural events and activities, and music and sports. On the other hand, more financially disadvantaged families are less able to invest time enriching their children, and they have less money to spend on cognitively stimulating materials and activities, high-quality child care, health care, and providing safe homes and neighborhoods [59–61]. Thus, children from lower-income families tend to experience lower quality home environments than children from higher-income families.

Furthermore, economic hardship also causes practical difficulties for parents because it is related to elevations in parental psychological distress. As mentioned earlier, lower SES is associated with poor parental mental health [62]. Parents’ psychological distress resulting from the experience of financial insecurity has been associated with greater conflict among family members and also with disrupted child-rearing skills, such as less positive parenting practices and more negative parenting practices (i.e., the adoption of unsupportive, uninvolved, and inconsistent parenting styles, and harsh disciplinary strategies). These parenting practices, in turn, are related to a range of damaging outcomes for children’s well-being [63–66]. SES could strongly influence the quality of communication and interactions between parents and children, and the higher quality of communications and interactions during early childhood have been positively associated with the later development of sociability and adaptability. On the other hand, the lower quality of communications and interactions during early childhood have been negatively associated with the later development of sociability and adaptability [53].

Further, the indirect psychosocial path involves the notion that not only being poor but also feeling of being poor in comparison to others (i.e., relative deprivation) elicits forms of psychological distress such as anxiety and psychological stress [67,68]. Relative deprivation is a social comparison theory that argues that individuals regularly compare themselves to those who are better off than they are [69]. Thus, it is likely that low SES partly influences child behavioral problems through upward comparisons of social class. The effects of stressors resulting from relative deprivation are particularly toxic for parents who are at the highest risk of experiencing socioeconomic hardship, as well as for those who are at an absolute economic disadvantage [70]. Such negative comparisons are likely to produce anxiety and psychological stress, which may negatively affect parent–parent relationships and parent-child interactions [71,72]. Thus, both absolute and relative detentions and economic disadvantages cause heightened economic stress, which in turn negatively affects parents’ parental psychological distress, which in turn negatively affects inter-familial conflict and parenting behavior, which in turn negatively affects children’s outcomes.

Considering the above situations, the findings of this study suggest that it is possible that family income influences all aspects of behavioral problems through several avenues, including the direct material path and the indirect psychosocial path.

When considering the impact of parental educational achievement on child outcomes in the current study, we also found that children of mothers who have lower educational achievement tend to have a higher risk of developing behavioral problems. This result is consistent with those of previous studies, which showed that mothers’ education is associated with externalized problems in offspring [73–75]. There may be several reasons children with lower-educated mothers have higher behavioral problems, including, experiencing less favorable parenting styles, lower-quality environments in the home, and exposure to stressful events in their surroundings. Further, less educated mothers tend to rely more on negative parenting attitudes, including physical and authoritarian disciplinary tactics [76–78]. It has been suggested that this is due to a lack of knowledge of means of developing a positive parental disposition toward children, of the counterproductive outcomes of severe disciplining measures, and of appropriate alternatives to physical and authoritarian disciplinary tactics [79]. In this study, although paternal educational level was significantly related to behavioral problems in the bivariate model, paternal educational level was not significantly related to behavioral problems in the multivariate model, which was mutually adjusted. Furthermore, path analyses also showed that paternal and maternal education levels were indirectly related to behavioral problems through family income. At the same time, maternal education level directly related to behavioral problems; on the other hand, paternal education level was not directly related to behavioral problems. One of the factors of the difference of effect level between paternal and maternal education levels may be the difference between the father and mother in spending time with their children. In this study, mean time spent talking or playing with children was 230.11 (SD = 146.45) minutes for mothers and 75.40 (SD = 77.25) minutes for fathers; mothers spent more time with their children than did fathers. The difference in the level of involvement may have led to the impact of maternal involvement on child outcomes.

In addition, we found maternal education to be an essential predictor of cognitively stimulating home environments, such as appropriate physical environments and learning experiences in the home, which influence children’s cognitive and behavioral outcomes [80,81]. Thus, the relationship between education and the qualities of home environments reveals the importance of disseminating information and knowledge concerning the development of positive parental dispositions toward children. Furthermore, there may be a specific possibility that Japanese women have higher relative deprivation in regard to education, which is in turn related to psychological distress.

While first-time upper secondary graduation rates in Japan are high for both men (96%) and women (98%), the share of female first-time graduates for all tertiary levels of education is the lowest in the OECD [26,27]. In addition, the gender gap in the Japanese labor market is among the largest in the OECD. The employment rate of tertiary-educated women between 25 and 64 years of age is 72% (below the OECD average of 80%), which is due to a significant proportion of women not being active in the labor market, while the employment rate of tertiary-educated men is 93% (above the OECD average of 88%). Even when they are employed, there are huge disparities between salaries for men and women with the same level of education. According to the Survey of Adult Skills, tertiary-educated men in Japan earn approximately 60% more than tertiary-educated women, the largest such gap in the OECD. Thus, Japanese women are likely to experience relative deprivation in regard to education and, thus, experience greater psychological stress, which in turn has a pathogenic influence on child-rearing skills. Thus, considering the above, the findings of this study also suggest that it is possible that maternal education level influences behavioral problems through several avenues, including the direct material path and the indirect psychosocial path.

Finally, regarding the impact of SES on child outcomes, there is another mechanism of the effects of low SES on behavioral problems in children: there is likely to be a role of genetic variation as a moderator. Socioeconomic circumstances are likely to influence child developmental outcomes through genetic factors. Children’s behavioral problems may be influenced by genetic risks, as well as their family’s environmental factors. A large body of evidence supports the conclusion that children’s behavioral problems are moderately heritable [82–85]. Several studies have suggested the extent to which children’s mental health functioning is affected by family environmental factors depends on genetic and early temperamental characteristics; environments help determine how genes express themselves [86–88]. Children with different genetic attributes will respond differently to the same environmental circumstances. In addition, epigenetic processes provide possibilities that low SES impacts health, including stress-related diseases, through environmental experiences that influence gene regulation [89,90]. Many studies suggest that exposure to adverse environments in early life has long-term consequences on later behavioral and neurobiological functioning including HPA axis reactivity to stress [91,92]. One possibility for the mechanism is that epigenetic alterations of the serotonin transporter gene expression account for the environmentally mediated effect of childhood victimization on HPA axis reactivity; childhood adversity experience may induce stable changes in HPA axis activity and increase vulnerability to psychopathology [93–95]. Exposure to adversity caused by low SES is likely to influence methylation; stressful life events increase vulnerability to affective disorders later in life, possibly mediated by methylation of the serotonin transporter gene [96,97]. Indeed, adults from secure family environments related to SES in early life had lower levels of depressive symptomology, whereas those who experienced insecure environments had higher levels of depressive symptoms [98–100]. Additionally, the impact of a stressful early family environment on depressive symptomatology has been found to be moderated by the serotonin transporter promoter polymorphism [98,99]. As mentioned earlier, children from low-SES families tend to be exposed to stressful life events through lower-quality home environments and lower quality of childcare (e.g., economic hardship and lower parents’ educational achievement). Thus, the influence of low SES on child behavior problems found in the present study could be influenced by genetic factors.

### Limitations

There are several limitations to this study, and these must be addressed in future research. First, the CBCL was completed by caregivers only, which likely introduced reporting bias. In addition, behavioral problems in school were not included. Teachers’ reports are needed to evaluate this more accurately; further explorations should combine teacher and caregiver CBCL ratings. Second, there are likely to be other factors that were not accounted for in our model. As mentioned earlier, there is a potential role of genetic variation as a moderator of the effects of low SES on behavioral problems in children. Several studies have suggested the extent to which children’s mental health functioning is affected by family environmental factors depends on genetic and early temperamental characteristics [86–88]. Future studies should investigate this possibility further to clarify more family environmental factors related to child mental health functioning, by using a genetically informative design (e.g., a twin or adoption study design), and estimating children’s early temperamental characteristics at baseline. Third, these findings may not be generalizable to all families, because there is a risk of attrition bias. The retention rate from T1 to T2 was 53.9%, and the returning participants in T2 tended to have relatively higher SES than the non-returning participants. There remains the possibility that our analysis could not fully evaluate the impact of SES on children outcomes, and our analysis may also underestimate the influence of SES. Future research would benefit from a study design that uses samples with higher retention rates (in particular, participants with lower SES). Finally, the sample was drawn from a limited geographical area in an urban metropolis in Japan. In this study, multivariate analyses revealed that lower maternal education level negatively influenced behavioral problems; on the other hand, paternal education level did not influence any behavioral problems. On the other hand, in research conducted in North America, lower paternal education level was found to be associated with increased mental health problems in the adult offspring of the fathers with lower education levels [99]. There is a possibility that the stronger impact of maternal education than paternal education on behavioral problems could be due to cultural effects of the status of mothers in Japan compared to western culture. Although Japanese and American women spend similar amounts of time on housework (excluding childcare), Japanese husbands spend less than a third of the time on household tasks than do American husbands [101]. Furthermore, similar discrepancies can be found with regard to child rearing. When comparing the average time spent per day with their young children, that of Japanese fathers is shorter than that of American and Swedish fathers; on the other hand, among these fathers, the Japanese work longer hours [102]. The difference in the level of involvement may have led to the influence on children outcomes. In addition, as mentioned earlier, the retention rate from the baseline survey to this survey was approximately 50%, and the returning participants tended to be relatively higher in SES than the non-returning participants. This indicates there is a risk of attrition bias. Therefore, there is the possibility that our analyses could not adequately evaluate the impact of SES on child outcomes, and our analyses may underestimate the influence of SES. Thus, the reproducibility of the current results should be confirmed using data from other regions in a variety of settings. Future research would benefit from samples with greater demographic and clinical diversity.

## Conclusion

The main finding of our study is that, for children, their family income and parental education situation at the time they enroll in preschool influences their outcomes after they enter in elementary school. In particular, after controlling for other SES indicators, low income and low maternal educational achievement were found to be significantly associated with mental health problems. Understanding the mechanisms of these associations could contribute to improvements in interventions aimed at preventing child maladjustment. The life course approach suggests that early exposure to adverse social circumstances leads to poor mental health in the future [103,104]. Our results indicate that we should be sensitive to social inequalities in children’s mental health problems and developmental outcomes, and should strive to reduce such inequalities. Further, in the long-term, we should focus not only on providing economic support, but also on education, as providing equal access to suitable educational opportunities can positively impact the next generation, and is likely to have a more permanent impact on the child-rearing environment than a temporary increase in income. Prior intervention trials, which have examined whether providing only additional income to families to address child health inequalities, have mainly had null effects; on the other hand, there are reported to be effective interventions that provide support with educational content [105]. A prior study also suggested that SES strongly influenced the quality of communication and interactions between parents and children and that positive communications and interactions during early childhood were associated with the higher development of sociability and adaptability later [53]. To prevent maladjustment of children in low-SES families, educational interventions for parents may contribute to the social adaptation of the child. In addition, disadvantaged youths tend to have lower rates of enrollment in senior high schools and college, and higher rates of dropout [106]. Consequently, if more parents can become better educated through an improved social system, it might lead to better developmental outcomes for children. Thus, both social scientists and welfare policymakers should focus on addressing both economic and educational aspects.

## Supporting information

S1 Fig Hypothesized model. (PDF)

S2 Fig Statistically significant paths for internalized behavioral problems. (PDF)

S3 Fig Statistically significant paths for externalized behavioral problems. (PDF)

S4 Fig Statistically significant paths for total behavioral problems. (PDF)

S1 Table Correlations among demographic characteristics, socioeconomic status, and behavioral problems. (DOCX)
