# Supplementary material for: Effect of socioeconomic status on behavioral problems from preschool to early elementary school – A Japanese longitudinal study
Source: PLoS One. 2018 May 24;13(5):e0197961. doi: 10.1371/journal.pone.0197961 (PMC5967727; doi:10.1371/journal.pone.0197961)
Supplement: S1 Fig — (PDF) [file pone.0197961.s001.pdf]

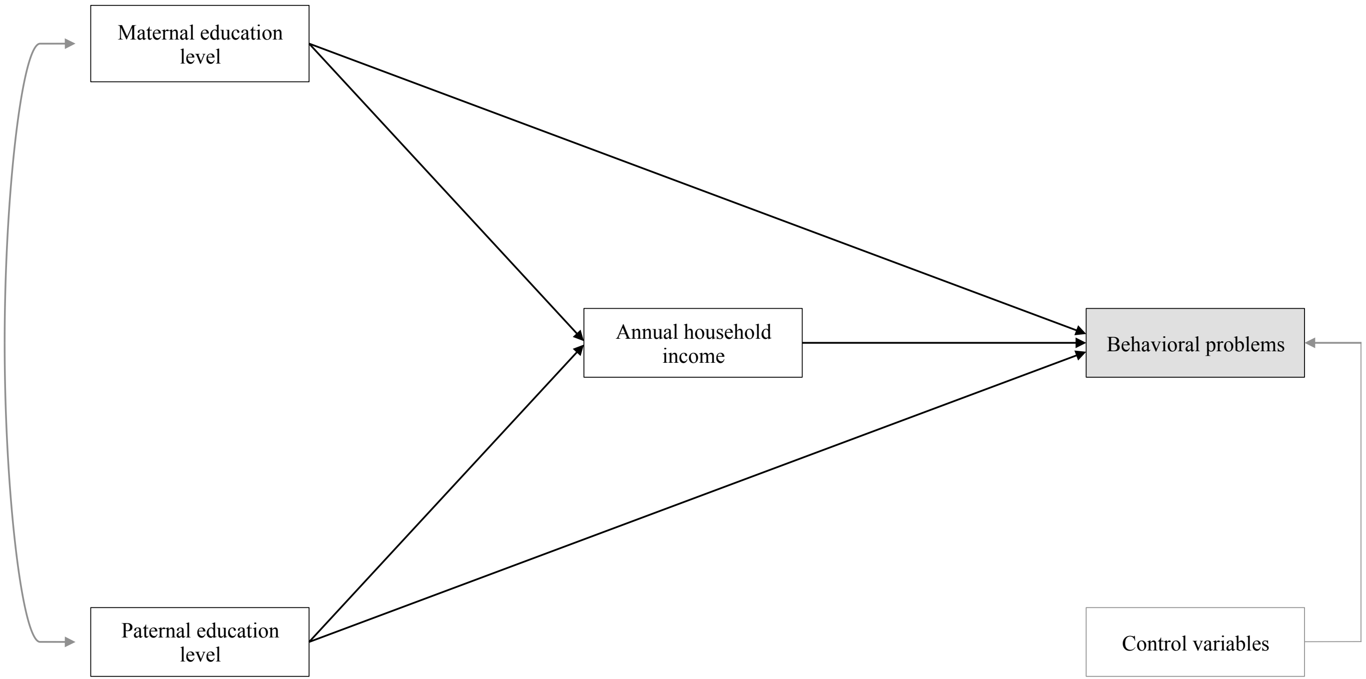

S1 Figure. Hypothesized model

Note: This model includes the hypothesized pathways among parental education levels, annual household income, and children's behavioral problems.
