# Supplementary material for: Effect of socioeconomic status on behavioral problems from preschool to early elementary school – A Japanese longitudinal study
Source: PLoS One. 2018 May 24;13(5):e0197961. doi: 10.1371/journal.pone.0197961 (PMC5967727; doi:10.1371/journal.pone.0197961)
Supplement: S4 Fig — (PDF) [file pone.0197961.s004.pdf]

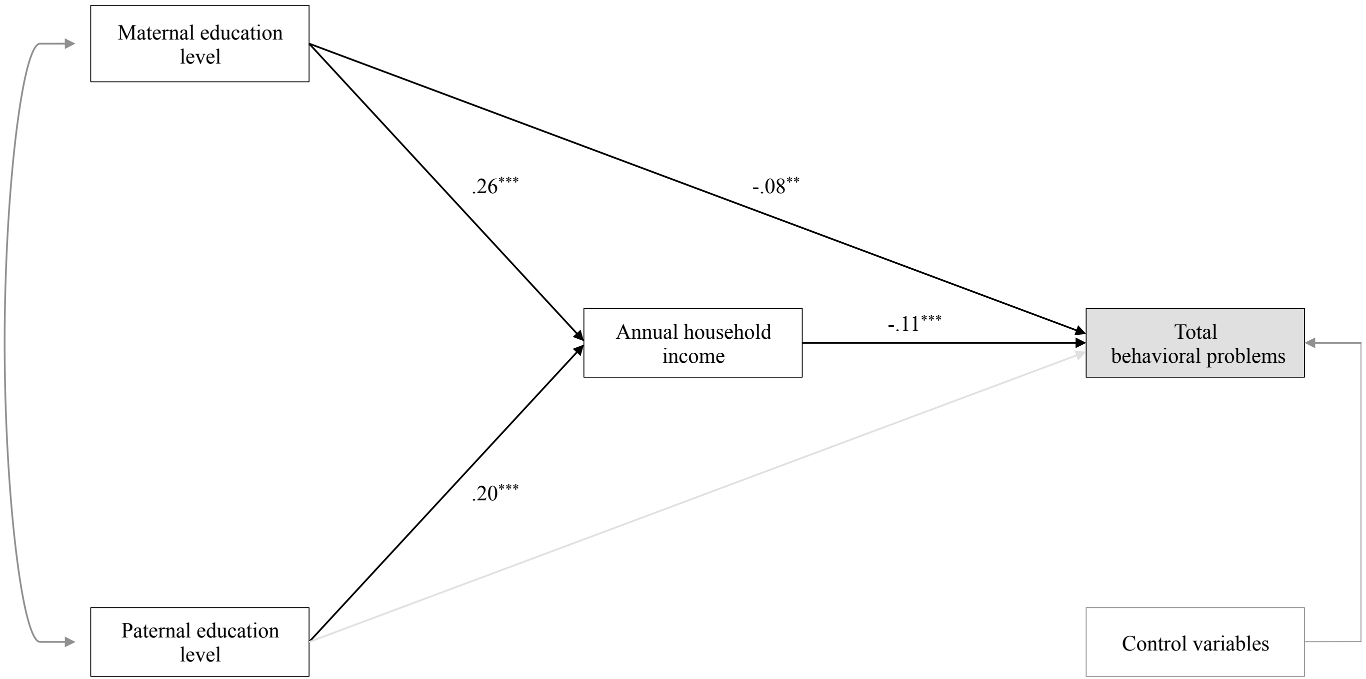

S4 Figure. Statistically significant paths for Total behavioral problems

Note: This model includes the paths that were statistically significant in the hypothesized model. All variables were converted to *z* scores. Standardized path coefficients are presented in the figure. Path analyses controlled for child sex and number of siblings (Variables significantly correlated with total behavioral problems in correlation analysis, were entered into the predictive model as control variables: *see S1 Table*).

Model fit statistics: χ^2 (7) = 14.54; CFI = .99; IFI = .99; RMSEA = .02

* p < .05; ** p < . 01; *** p < .001
