# Supplementary material for: Effect of socioeconomic status on behavioral problems from preschool to early elementary school – A Japanese longitudinal study
Source: PLoS One. 2018 May 24;13(5):e0197961. doi: 10.1371/journal.pone.0197961 (PMC5967727; doi:10.1371/journal.pone.0197961)
Supplement: S1 Table — (DOCX) [file pone.0197961.s005.docx]

| **S1 Table. Correlations among demographic characteristics, socioeconomic status, and behavioral problems (*n* = 1,712)** | | | | | | | | | | | |
| --- | --- | --- | --- | --- | --- | --- | --- | --- | --- | --- | --- |
| *Variable* | *1* | *2* | *3* | *4* | *5* | *6* | *7* | *8* | *9* | *10* | *11* |
| Demographics |  |  |  |  |  |  |  |  |  |  |  |
| 1. Child sex | ― |  |  |  |  |  |  |  |  |  |  |
| 2. Family composition | .03 | ― |  |  |  |  |  |  |  |  |  |
| 3. Family status | .02 | .12^***^ | ― |  |  |  |  |  |  |  |  |
| 4. Number of siblings | .04 | .05^*^ | .13^***^ | ― |  |  |  |  |  |  |  |
| 5. Preschool institution attended | .01 | .05^*^ | .10^***^ | .04 | ― |  |  |  |  |  |  |
| Socioeconomic status |  |  |  |  |  |  |  |  |  |  |  |
| 6. Annual household income | -.03 | -.08^**^ | -.29^***^ | -.04 | -.02 | ― |  |  |  |  |  |
| 7. Maternal education level | -.02 | -.05^*^ | -.11^***^ | -.06^*^ | -.06^*^ | .34^***^ | ― |  |  |  |  |
| 8. Paternal education level | -.00 | -.08^**^ | -.06^*^ | -.03 | -.15^***^ | .29^***^ | .41^***^ | ― |  |  |  |
| Behavioral problems |  |  |  |  |  |  |  |  |  |  |  |
| 9. Internalized behavioral problems | .01 | .01 | .01 | .09^***^ | .00 | -.13^***^ | -.12^***^ | -.07^**^ | ― |  |  |
| 10. Externalized behavioral problems | .13^***^ | .03 | .06^*^ | .01 | .04 | -.12^***^ | -.12^***^ | -.10^***^ | .53^***^ | ― |  |
| 11. Total behavioral problems | .07^**^ | .02 | .04 | .05^*^ | .03 | -.15^***^ | -.14^***^ | -.10^***^ | .86^***^ | .88^***^ | ― |
| *Note*: [Demographics] Child sex (0 = female, 1 = male), Family composition (0 = nuclear family, 1 = expanded family), Family status (0 = two parents, 1 = one parent), Number of siblings (0 = one or more siblings, 1 = no siblings), Preschool institution attended (0 = kindergarten, 1 = nursery school). [Socioeconomic status] Annual household income (1 = <3 million JPY, 2 = 3-4 million JPY, 3 = 5-6 million JPY, 4 = ≥7 million JPY), Maternal education level (1 = compulsory education, 2 = upper secondary school, 3 = less than four years at college/university, 4 = over four years at college/university), Paternal education level (1 = compulsory education, 2 = upper secondary school, 3 = less than four years at college/university, 4 = over four years at college/university). [Behavioral problems] Behavioral problems were assessed using the Child Behavior Checklist/4–18 (CBCL).  ^*^*p* < .05; ^**^*p* < .01; ^***^*p* < .001 | | | | | | | | | | | |
